# Topical Chlormethine Gel in the Treatment of Lymphomatoid Papulosis: A Case Report and Literature Review

**DOI:** 10.3390/jcm14155338

**Published:** 2025-07-29

**Authors:** Miriam Teoli, Martina Caviglia, Federica Rega, Luca Barbieri, Marco Ardigò, Victor Desmond Mandel

**Affiliations:** 1 Porphyria and Rare Diseases Unit, San Gallicano Dermatological Institute-IRCCS, 00144 Rome, Italy; miriam.teoli@ifo.it (M.T.); luca.barbieri@ifo.it (L.B.); 2 Dermatologic Unit, Department of Clinical Internal, Anesthesiologic and Cardiovascular Sciences, La Sapienza University of Rome, 00185 Rome, Italy; marticav8@gmail.com (M.C.); rega.federica@alice.it (F.R.); 3 Dermatology Unit, Humanitas Research Hospital-IRCCS, 20089 Rozzano, Italy; ardigo.marco@gmail.com

**Keywords:** lymphomatoid papulosis, cutaneous T-cell lymphoma, mechlorethamine, chlormethine, nitrogen mustard

## Abstract

**Background**: Lymphomatoid papulosis (LyP) is a primary cutaneous CD30-positive T-cell lymphoproliferative disorder presenting with self-healing erythematous papulonodular lesions that may ulcerate and scar. Treatment varies by lesion extent, location, and severity. **Case Report**: We describe a 57-year-old man with acral LyP successfully treated with chlormethine gel (CG). The patient experienced impaired second finger mobility for over 3 months due to an ulcerated nodular mass. After 3 months of CG treatment, complete remission, symptom resolution, and full joint recovery were achieved. Six months post-treatment, the patient remained in remission. **Conclusions**: This case underscores the effectiveness of CG in achieving sustained remission in acral LyP, suggesting its potential as a treatment option for this rare condition.

## 1. Introduction

Lymphomatoid papulosis (LyP) is a cutaneous CD30-positive T-cell lymphoproliferative disorder (CD30+ TLPD), a subcategory of cutaneous T-cell lymphoma that mostly has a non-aggressive course and accounts for about 15% of all cutaneous lymphomas [1]. Its etiology and pathogenetic mechanisms are currently unknown. The disease exhibits two peak frequencies: first, in children under 18 years of age, and second, in adults between the fourth and fifth decades of life [2]. The clinical spectrum of LyP includes self-healing erythematous papulonodular lesions that often become pustular, ulcerated, and resolve with scarring. These lesions can be single or multiple and commonly occur on the trunk and limbs [3]. Rare cases of segmental or localized presentations involving acral and facial sites have been described [4,5,6,7]. Acral LyP, in particular, is an uncommon variant that can lead to significant morbidity due to pain, ulceration, or impaired mobility. Its rarity and clinical impact underscore the importance of individualized management strategies.

LyP may be preceded, associated, or followed by other lymphatic neoplasms, with mycosis fungoides (MF) being the most commonly associated secondary lymphoma [8,9]. Treatment outcomes are unsatisfactory and may vary depending on the extent, location, and severity of the skin lesions. A ‘watchful waiting strategy’ approach can be considered for patients with a single or relatively few lesions. However, topical therapies such as corticosteroids or chlormethine gel may be preferred. In particular, chlormethine gel offers a non-steroidal, skin-directed approach with a long history of safe use in cutaneous lymphomas, and may be advantageous in acral locations where minimizing tissue atrophy and preserving function are priorities [7]. Cases with disseminated disease may benefit from phototherapy or methotrexate, which has been specifically recommended in treatment guidelines such as those by the Dutch Cutaneous Lymphoma Group [10]. Broader guidance for CD30+ lymphoproliferative disorders, including LyP, is also provided in the EORTC, ISCL, and USCLC consensus recommendations [11]. Alternative therapeutic approaches include antibiotics, antihistamines, oral steroids, or brentuximab, a monoclonal antibody targeting CD30 (Table 1) [2,3,4,5,6,7,8,9,10,11,12].

Herein, we report a rare case of LyP with exclusively acral localization treated with commercially available chlormethine gel (CG). Additionally, we provide a brief review of the related literature.

## 2. Case Report

**Figure 1:**
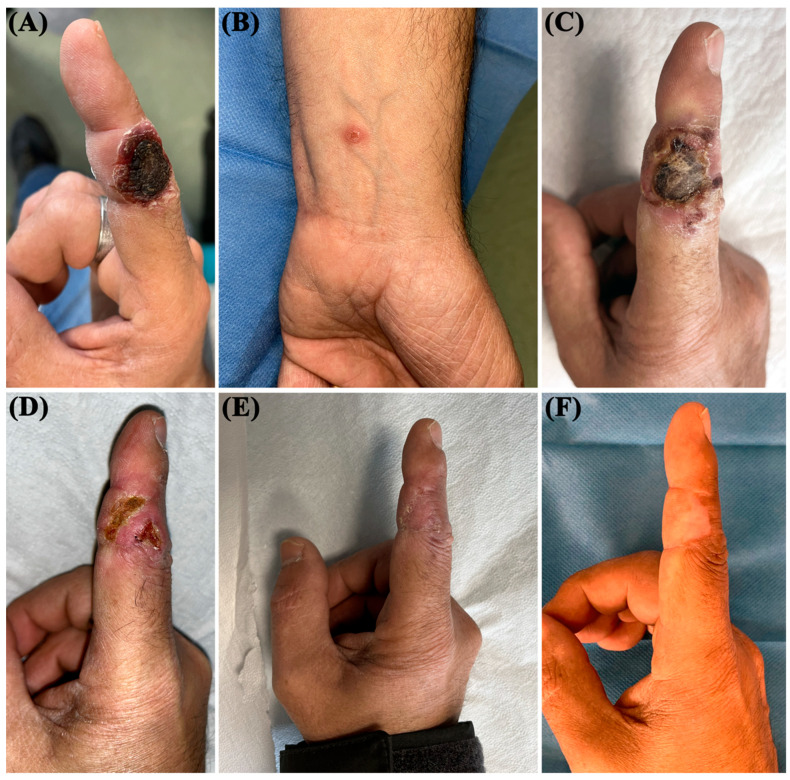
Clinical history of the patient. (**A**) Baseline presentation: Ulcerative nodular mass (2 × 1.5 cm) with raised edges on the second finger of the right hand, causing impaired joint mobility. (**B**) Additional lesion: Small erythematous papule (0.5 × 0.4 cm) observed on the left wrist. (**C**) Post-curettage view of the second finger lesion, prior to biopsy. (**D**) At 1 month after CG initiation: Partial regression of the nodular lesion with visible reduction in ulceration and inflammation. (**E**) At 3 months after CG initiation: Complete clinical remission of finger and wrist lesions, with full recovery of joint function. (**F**) At 6-month follow-up: Sustained remission with no recurrence after CG discontinuation.

**Figure 2:**
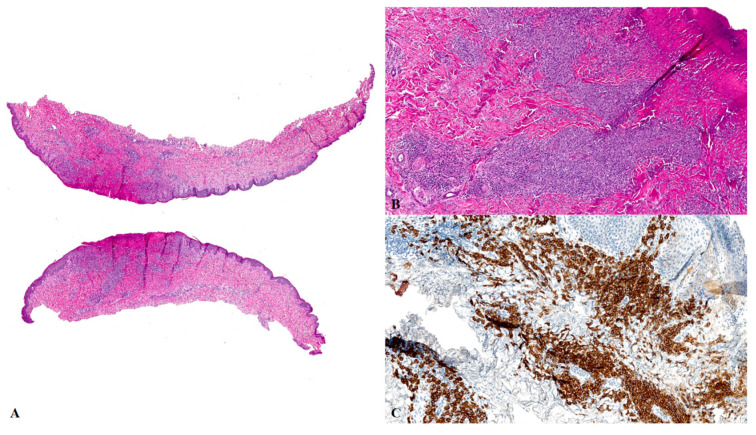
Cutaneous biopsy specimen of the ulcerative nodular lesion of the second finger of the right hand. (**A**) Perivascular lymphocytic infiltrate with V-shape arrangement [hematoxylin-eosin (HE); original magnification: 40×]. (**B**) The infiltrate is composed mainly by medium-sized cells, with convoluted nuclei, associated with sparse histiocytes and eosinophils (HE; original magnification: 200×). (**C**) The larger cells are diffusely and strongly positive for CD30 (CD30 stain; original magnification: 200×).

A 57-year-old man, a painter by profession, was referred to our Porphyria and Rare Disease Unit in December 2022 for an evaluation of a painful lesion on the second finger of his right hand that had persisted for more than 3 months. He complained of impaired mobility of the finger joint, which prevented him from working. The patient had previously been evaluated at another center, where no specific diagnosis or treatment had been initiated. His family and medical history were unremarkable, and he was not taking any medications. A clinical examination revealed an ulcerative nodular mass measuring 2 × 1.5 cm, with well-defined and raised edges, located on the second finger of the right hand (Figure 1A). The lesion was covered by a strongly adherent eschar that was firm on palpation. A small (0.5 × 0.4 cm) erythematous papule was also observed on the left wrist (Figure 1B). We performed a complete routine laboratory investigation, including immunologic tests (immunoglobulins, rheumatoid factor, antinuclear factor, and organ-specific antibodies), and all values were within the normal range. To exclude systemic involvement, the patient also underwent an ultrasound examination of superficial lymph node sites, abdominal ultrasound, and a chest X-ray—all of which were unremarkable. After curettage (Figure 1C), an 8 mm punch biopsy was performed on the ulcerative nodular lesion of the second finger. A histopathologic analysis revealed a dense dermal lymphoid infiltrate composed of CD3+, CD4−, and CD8+ elements, along with clusters of CD30+ lymphomatoid cells (Figure 2). These features were suggestive of a type E LyP, characterized by the presence of angiotropism/angiodestruction with perivascular and intraluminal infiltrates of atypical cells, where T cells can be either CD4+ or CD8+. Additional proliferation markers such as Ki-67 were not deemed necessary, as the immunophenotype and T-cell receptor gene rearrangement analysis confirming monoclonality were sufficient to establish the diagnosis. A T-cell receptor gene rearrangement analysis showed monoclonality, and the histopathologic features were consistent with a diagnosis of LyP. Due to the painful symptoms and associated functional impairment, topical treatment with CG was prescribed. The patient applied a thin layer of CG to the lesion on the second finger every other day during the first week of treatment and then once daily in the evening, as recommended to reduce the risk of phototoxicity and enhance local tolerability. After approximately two weeks of CG application, the patient developed local irritative contact dermatitis (ICD), presenting with burning and itching sensations. Topical clobetasol propionate cream was initiated within a few days of symptom onset, and CG application was temporarily reduced. As corticosteroid treatment began shortly after the appearance of symptoms and was effective in relieving the reaction, the precise duration of the ICD could not be determined; this event did not recur. Once the irritation was resolved, the frequency of CG application was gradually increased, and the topical steroid was discontinued. One month later, a favorable response was observed (Figure 1D). A complete clinical remission of the lesions, with the resolution of painful symptoms and the fully functional recovery of the finger joint, was observed after 2 more months (Figure 1E). No serious adverse effects were reported during CG application, and the treatment was discontinued in March 2023. To maintain complete remission, methotrexate (7.5 mg per week) was prescribed in combination with folic acid (5 mg per week) following recommendations from the Dutch Cutaneous Lymphoma Group for the management of recurrent or symptomatic LyP [10]. Meanwhile, the erythematous papule observed on the left wrist resolved spontaneously. The patient has continued monthly follow-ups, including laboratory assessments of liver and kidney function as well as blood counts. At 6 months after the discontinuation of CG application, the patient remains in complete remission, maintained with methotrexate and folic acid.

## 3. Discussion

LyP shares many therapeutic options with other TLPDs, such as MF. However, no curative therapy is currently available. Guideline-based approaches emphasize treatment individualization according to lesion number, location, and severity. Methotrexate is supported as a first-line systemic option for widespread disease by the Dutch Cutaneous Lymphoma Group, while the EORTC/ISCL/USCLC consensus offers broader recommendations on management and clinical endpoints for CD30+ disorders [10,11]. Chlormethine gel was selected as monotherapy based on the localized nature of the lesions and the absence of systemic involvement. In line with the clinical review by Martinez-Cabriales et al. [7], topical monotherapy is an appropriate and effective first-line approach for patients with limited disease. The decision to avoid combination therapy with corticosteroids or phototherapy was guided by the desire to minimize the treatment burden, reduce potential cumulative side effects, and achieve a targeted, skin-directed intervention in a functionally sensitive area. The patient’s complete remission and functional recovery without the need for adjunctive treatments support the adequacy of monotherapy in this context [10,11]. Chlormethine, also known as mechlorethamine, is a nitrogen mustard classified as an alkylating agent. It binds to the N7 nitrogen of the base guanine in DNA, crosslinks two DNA strands, and inhibits rapidly proliferating cells, thereby preventing cell duplication [13]. CG (chlormethine 0.016% *w*/*w*, equivalent to 0.02% chlormethine hydrochloride) was approved by the US Food and Drug Administration in 2013 for treating stage IA and IB MF in patients who had previously received skin-directed therapies. In contrast, the European Medicines Agency approved its use for treating adult patients with MF in 2017 [13]. CG can be used as a monotherapy, in combination with systemic therapy, and as maintenance treatment [13,14]. Although the CG label recommends daily application, the frequency can be reduced to mitigate skin reactions.

In our case, we followed the same dosing schedule used for the MF treatment, achieving rapid and complete remission. Furthermore, the patient experienced a rapid and complete recovery of the finger joint functionality. The only side effect observed was moderate ICD, which is the most common skin reaction associated with CG therapy. However, ICD was effectively controlled with topical steroid use, improving the tolerability of CG, as reported in real-life experiences with MF treatment [14]. The long-term effects of CG have not been evaluated because of the short follow-up period available. Recently, Dege et al. described the use of CG in an 80-year-old patient with coexisting MF and LyP, observing the resolution of LyP papules after the localized application of the gel, which had been initiated primarily for MF lesions in adjacent areas [15]. Although their case provides relevant clinical insight into chlormethine use in LyP, it involved mixed disease and treatment not specifically tailored to LyP from the outset. In contrast, our report presents the first case of exclusive acral LyP successfully treated with chlormethine gel as monotherapy, initiated specifically to target LyP lesions and independent of concurrent MF. Additional cases support the use of topical alkylating agents for LyP. Shang et al. reported successful management of regional LyP with nitrogen mustard solution combined with interferon-alpha-2b [16]. Also, the association between LyP and other TLPDs is well known. Trager et al. described 10 patients with MF who developed LyP after experiencing an important inflammation during nitrogen mustard treatment [17]. The role of proliferation signals as chronic drug-induced inflammation has been hypothesized, but the triggering event leading to proliferation remains unclear [7]. Therefore, future prospective studies are warranted to evaluate the potential long-term side effects of CG and to investigate any possible correlation between CG-induced local inflammation and cutaneous T-cell lymphoma. The present case is limited by its single-patient design and a relatively short follow-up duration (6 months), which does not allow for conclusions about long-term safety or recurrence. Broader clinical experience and longer-term data are needed to confirm the durability of remission and establish the optimal role of CG in LyP management.

## 4. Conclusions

Our case highlights the importance of personalized treatment in minimizing the disease severity and achieving early complete remission. CG has proven to be a safe, fast-acting, easy-to-apply drug with excellent cosmetic results in LyP. Therefore, CG should be considered a viable therapeutic option for managing LyP, particularly in localized presentations. However, further studies are needed to validate these findings, assess long-term outcomes, and better define its role in clinical practice.

## Figures and Tables

**Table 1:** Summary of therapeutic options for lymphomatoid papulosis.

Therapy Type	Treatment Options	Indications	Advantages	Limitations
Observation	Watchful waiting	Few asymptomatic lesions	Non-invasive; avoids overtreatment	Risk of recurrence; no lesion control
Topical therapies	Chlormethine gel Topical corticosteroids	Localized lesions (1–3 papules), especially acral/facial	Skin-directed, low systemic risk; chlormethine gel offers cytotoxic effect	Irritant dermatitis (chlormethine gel); atrophy with prolonged steroids
Phototherapy	PUVA, narrowband UVB	Multifocal lesions or widespread involvement	Non-invasive; widely available	Requires frequent sessions; impractical for limited disease
Systemic therapies	Methotrexate (low dose) Oral corticosteroids	Recurrent, symptomatic, or disseminated disease	Effective in maintenance; methotrexate well-tolerated	Immunosuppression; lab monitoring needed
Other treatments	Interferon-α Brentuximab vedotin Antibiotics/antihistamines (supportive)	Severe or treatment-resistant cases symptom management	Targeted therapy options available	Limited data; potential toxicity; often off-label

## Data Availability

All authors had full access to all of the data in this study and take complete responsibility for the integrity of the data and the accuracy of the data analysis. The data that support the findings of this study are available from the corresponding author upon reasonable request.
